# Integrated Institutional Transformation Theory (IITT): Toward an Institutional Theory for the Transformation of Primary Healthcare Centers Through the Tikena17 Conceptual Framework

**DOI:** 10.7759/cureus.116222

**Published:** 2026-09-14

**Authors:** Ahmed Khalafalla Abdalla

**Affiliations:** 1 Family Medicine, Tabuk Health Cluster, Tabuk, SAU

**Keywords:** healthcare value, health-system integration, institutional transformation, primary healthcare center (phc), system thinking, tikena17 institutional transformation framework

## Abstract

Primary healthcare centers are fundamental to healthcare systems, yet sustainable institutional transformation remains constrained by fragmented governance, disconnected institutional structures, and the absence of an integrated institutional perspective, as existing frameworks largely address governance, institutional performance, quality improvement, integrated care, and institutional learning as separate domains rather than components of a unified institutional system. This study proposes the Integrated Institutional Transformation Theory (IITT) as a theoretical perspective for explaining institutional transformation in primary healthcare centers and introduces the Tikena17 Institutional Transformation Framework (Tikena17-ITF) as its conceptual representation. A conceptual theory development methodology was employed using a critical integrative review of institutional management and healthcare management literature, wherein systems thinking principles were applied to synthesize major theoretical traditions into a proposed integrated perspective on institutional transformation, followed by the development of a conceptual framework and a set of theoretical propositions. The proposed theory conceptualizes institutional transformation as a dynamic systemic process emerging from the interaction of interconnected institutional components rather than isolated institutional functions. Consistent with this perspective, the Tikena17-ITF represents institutional transformation through five interconnected layers comprising 17 institutional components. The proposed theory and framework offer a conceptual foundation for understanding institutional transformation in primary healthcare centers and for generating hypotheses that can be examined through future empirical research, implementation studies, institutional assessment, and validation across diverse healthcare settings. Their potential contribution to healthcare value, institutional reliability, accountability, maturity, and long-term sustainability remains subject to empirical evaluation.

## Introduction

Primary healthcare centers constitute a cornerstone of contemporary healthcare systems and are widely recognized as fundamental to achieving universal health coverage, improving population health, and promoting equity, efficiency, and sustainability [1,2]. However, sustainable institutional transformation remains challenging because healthcare organizations must simultaneously address governance, institutional planning, organizational structures, processes, human capabilities, performance, and models of care, which are often managed through fragmented and insufficiently integrated approaches [3-6]. As healthcare systems undergo profound demographic, epidemiological, technological, and societal changes, increasing attention has therefore shifted from improving isolated services toward strengthening healthcare institutions as adaptive systems capable of continuous learning, resilience, and sustainable performance [1-3,7,8]. Primary healthcare centers have particularly broad institutional responsibilities, encompassing health promotion, disease prevention, chronic disease management, care coordination, and continuity of care [1,2]. Consequently, institutional effectiveness depends not only on clinical service delivery but also on the ability to govern effectively, plan strategically, develop organizational structures and capabilities, manage performance, support organizational learning, and sustain appropriate models of care [4,7,9,10]. Over recent decades, healthcare management literature has generated numerous conceptual models and frameworks addressing governance, quality management, organizational transformation, institutional performance, integrated care, risk management, organizational learning, high-reliability organizations (HROs), and accountable care organizations (ACOs) [3-8,10-13]. These contributions have substantially advanced knowledge and provided valuable approaches for addressing specific institutional domains. Nevertheless, the conceptual limitation identified in this study is not the absence of frameworks addressing these domains, but their limited integration into a coherent explanation of institutional transformation as a system-level phenomenon. Existing approaches differ in scope, purpose, and level of analysis, and many primarily focus on particular institutional functions or transformation dimensions rather than explicitly explaining how multiple institutional components interact and collectively shape institutional transformation [4-8,12,13]. This fragmentation may make it difficult to understand how changes in governance, structures, capabilities, relationships, operational flows, performance, learning, reliability, accountability, and sustainability are interconnected within the same institutional system. In this study, institutional transformation is defined as a dynamic and sustained process through which interconnected institutional structures, capabilities, relationships, processes, and outcomes are progressively reconfigured to strengthen the institution's capacity to create healthcare value, achieve reliable performance, adapt to change, and sustain its intended mission. From this perspective, institutional transformation is not considered synonymous with organizational restructuring, performance improvement, quality improvement, or care integration alone; rather, these represent interacting dimensions of a broader institutional transformation process. This conceptual gap provides the rationale for proposing the Integrated Institutional Transformation Theory (IITT) as a theoretical perspective for explaining institutional transformation in primary healthcare centers. IITT proposes that healthcare institutions can be understood as integrated institutional systems in which multiple institutional components continuously interact rather than operate as isolated managerial functions. The Tikena17 Institutional Transformation Framework (Tikena17-ITF) is introduced as the conceptual representation of this proposed theory, organizing these interacting institutional components into a structured architecture. The study therefore aims to provide a coherent theoretical foundation for understanding the relationships among institutional components and to generate theoretical propositions that can guide future empirical testing, implementation research, and the development of institutional assessment approaches.

## Technical report

Objective

This study proposes the IITT as a theoretical perspective for explaining institutional transformation, with primary healthcare centers serving as the primary context for its conceptual development, and introduces the Tikena17-ITF as the conceptual representation of the proposed theory. While the theory is developed in the context of primary healthcare, its potential applicability to other healthcare institutions is proposed as a subject for future empirical investigation rather than as an established generalization.

Materials and methods

The present study employed a conceptual theory development methodology based on a critical integrative review of institutional management and healthcare management literature. Unlike empirical studies that generate primary data, conceptual research seeks to synthesize existing theoretical knowledge to develop new explanatory frameworks and theoretical models [8]. The methodological process was designed to move systematically from the identification of relevant theoretical domains to conceptual synthesis, integration, and theory formulation. The literature was purposively identified and selected according to its conceptual relevance to institutional transformation in healthcare, with particular attention to theoretical and conceptual contributions addressing primary healthcare systems, institutional governance, integrated care, organizational transformation, institutional performance, organizational learning, HROs, ACOs, and healthcare value [1,3,5-7,12,13]. Literature was considered relevant when it contributed concepts, constructs, relationships, or theoretical principles that could inform the understanding of institutional transformation or the interaction among institutional domains. The purpose of the review was therefore not to estimate an empirical effect or provide a systematic quantitative synthesis, but to identify and critically examine complementary theoretical perspectives relevant to the development of an integrated institutional theory. Systems thinking was adopted as the primary analytical perspective for examining relationships among institutional domains and identifying structural interactions that existing frameworks often address independently [9]. Concepts and constructs relevant to institutional transformation were extracted from the reviewed literature and organized according to their institutional meaning, theoretical domain, and potential relationships with other concepts. The extracted concepts were then critically compared to identify areas of conceptual convergence, complementarity, overlap, and fragmentation across the reviewed theoretical traditions. The development of the IITT followed an iterative conceptual synthesis process comprising four sequential but interconnected stages. First, the principal theoretical domains influencing institutional transformation were identified from the relevant literature. Second, the concepts and relationships within these domains were critically examined to identify conceptual boundaries, complementarities, overlaps, and limitations in existing approaches. Third, complementary concepts were integrated using systems thinking to develop a coherent institutional perspective in which institutional domains were conceptualized as interconnected rather than isolated functions. Fourth, the resulting conceptual structure was organized into the Tikena17-ITF as the conceptual representation of IITT, and theoretical propositions were formulated to express the principal relationships proposed by the theory. The five layers of the Tikena17-ITF were derived by grouping conceptually related institutional domains according to their function and position within the proposed transformation architecture. The 17 components were subsequently derived by further disaggregating these domains into distinct but interconnected institutional constructs represented in the reviewed literature. Components were retained when they demonstrated clear conceptual relevance to institutional transformation and contributed a distinct function within the integrated institutional system. The resulting architecture was reviewed iteratively to ensure conceptual coherence, minimize unnecessary duplication among components, and preserve the proposed relationships among institutional domains.

The nine theoretical propositions were derived from the principal relationships identified through the conceptual synthesis. Each proposition expresses a theoretically proposed relationship among interconnected institutional domains or between institutional integration and proposed transformation-related outcomes. These propositions are therefore presented as theoretically derived hypotheses for future empirical examination rather than as empirically demonstrated relationships.

This methodological sequence can be summarized as: relevant literature → theoretical domains → concept extraction → critical comparison → systems-based synthesis → five layers → 17 components → nine theoretical propositions → IITT and Tikena17-ITF. Because this study is entirely conceptual and does not involve human participants, patient data, biological specimens, or experimental interventions, ethical approval and informed consent were not required.

Literature review

Institutional Transformation in Primary Healthcare

Primary healthcare centers have undergone substantial transformation over recent decades, evolving from providers of essential healthcare services into institutions responsible for population health management, health promotion, disease prevention, and coordination across healthcare systems. The World Health Organization (WHO) consistently recognizes primary healthcare as the foundation for achieving universal health coverage, improving health outcomes, and promoting equity, efficiency, and sustainability [1]. This transformation reflects a broader shift in the role of healthcare institutions, in which institutional success increasingly depends not only on service delivery capacity but also on the ability to manage change, integrate organizational capabilities, and generate sustainable healthcare value [10,11]. These developments suggest that institutional transformation extends beyond individual improvement initiatives and involves changes across multiple interconnected institutional domains.

Primary Healthcare and Integrated Care

Integrated care has become a dominant paradigm in contemporary healthcare reform, seeking to coordinate healthcare delivery across multiple levels of care to improve continuity, patient experience, and health outcomes [3,10]. The literature indicates that integrated care requires more than redesigned clinical pathways and depends on effective governance, institutional leadership, integrated information systems, supportive organizational cultures, and coordinated organizational processes [10,11]. Thus, integrated care provides an important perspective on the coordination of healthcare delivery, while also demonstrating that successful care integration is influenced by wider institutional conditions. However, integrated care frameworks primarily address coordination and integration of care rather than providing a general theoretical explanation of how the broader institutional system transforms over time.

Institutional Governance

Institutional governance has become a central concept in contemporary management literature through its emphasis on accountability, transparency, strategic decision-making, stakeholder relationships, and risk oversight [10,11]. Within healthcare, governance has expanded to include corporate governance, clinical governance, digital governance, and data governance. Governance frameworks therefore provide important explanations of how institutions are directed, controlled, and held accountable. Their primary analytical focus, however, remains governance and oversight, providing less explicit explanation of how governance interacts dynamically with organizational structures, capabilities, operational processes, learning, reliability, and other institutional domains to constitute an integrated transformation process.

Organizational Transformation

Organizational transformation literature has significantly contributed to understanding change management, organizational development, restructuring, and leadership [12]. These perspectives are particularly valuable for explaining how organizations respond to environmental pressures and implement major organizational change. Nevertheless, transformation models have often conceptualized transformation through the lens of change programs, organizational development, or management initiatives. This emphasis provides important insights into the dynamics of change but offers a more limited explanation of transformation as a continuous institutional process emerging from interactions among multiple organizational and institutional components.

Institutional Performance

Institutional performance has evolved from measuring operational efficiency toward evaluating an organization's capability to achieve strategic objectives and generate societal value [2,12]. Performance frameworks have therefore made substantial contributions to measurement, accountability, and strategic management. However, performance is frequently represented primarily as an outcome or measurement domain rather than as a phenomenon that may emerge from interactions among governance, leadership, organizational culture, institutional capabilities, and learning processes. Consequently, performance frameworks provide important mechanisms for assessing institutional results but offer a more limited theoretical account of how interconnected institutional conditions collectively shape transformation.

Learning Organizations

Learning organization theory emphasizes continuous learning as a source of organizational adaptation, innovation, resilience, and sustained institutional performance [4]. This perspective contributes an important explanation of how organizations develop the capacity to learn from experience and adapt to changing circumstances. Nevertheless, organizational learning has generally been conceptualized as a distinct organizational capability rather than as one component of a broader institutional architecture systematically connected with governance, organizational structure, operational processes, performance, healthcare delivery, and value creation.

High-Reliability Organizations

HRO theory has advanced understanding of organizations operating in complex and high-risk environments by emphasizing organizational mindfulness, resilience, risk management, and failure prevention [5,13]. These principles have substantially influenced patient safety and healthcare quality. HRO theory therefore provides an important explanation of how institutions can maintain reliable performance under conditions of complexity and risk. Its principal focus, however, is operational reliability and resilience, rather than the broader institutional transformation process through which governance, structure, capabilities, learning, performance, accountability, and sustainability may become progressively integrated.

Accountable Care Organizations

ACOs introduced a management paradigm linking healthcare quality, patient outcomes, resource efficiency, and institutional accountability [6,13]. This model has significantly advanced approaches to integrated healthcare delivery by connecting clinical outcomes with accountability and resource management. However, its primary emphasis is on care coordination, outcomes, and accountability mechanisms rather than on providing a comprehensive theoretical explanation of the institutional relationships and capabilities through which sustainable institutional transformation may occur.

Comparative synthesis and conceptual gap

Taken together, these theoretical traditions provide substantial and complementary explanations of important dimensions of healthcare institutional development. Integrated care emphasizes coordination across services; governance emphasizes direction, oversight, and accountability; organizational transformation emphasizes change and adaptation; performance frameworks emphasize measurement and strategic outcomes; organizational learning emphasizes adaptation through learning; HRO theory emphasizes reliability and resilience; and accountable care emphasizes the integration of outcomes, resource use, and accountability [3-6,10-13]. Their contributions are therefore not mutually exclusive, and several concepts overlap with dimensions incorporated within the proposed IITT. The conceptual limitation identified in this study lies instead in the level of integration among these domains. Existing perspectives generally originate from different theoretical traditions and address particular institutional functions, outcomes, or transformation mechanisms. Consequently, they provide valuable explanations of individual dimensions but offer less explicit theoretical specification of how these dimensions interact as parts of a single institutional system and how such interactions may collectively constitute institutional transformation. The gap is therefore not a lack of knowledge about individual institutional domains, but the absence of a sufficiently integrated theoretical account that places their interactions at the center of the explanation. This synthesis provides the rationale for proposing the IITT. IITT does not seek to replace existing theories or frameworks; rather, it proposes an integrative theoretical perspective in which their relevant institutional constructs can be understood as interconnected dimensions of a broader institutional transformation process. The Tikena17-ITF subsequently represents this proposed perspective by organizing the interconnected institutional domains into a coherent conceptual architecture. The relationships proposed within this architecture are theoretical propositions requiring empirical examination rather than established causal relationships.

Critical synthesis of the literature

The reviewed literature demonstrates substantial progress in the conceptualization of governance, primary healthcare, integrated care, organizational transformation, institutional performance, organizational learning, reliability, and accountability. These theoretical traditions have made important and complementary contributions to understanding how healthcare organizations can be governed, transformed, coordinated, evaluated, adapted, and sustained. Rather than indicating a lack of relevant theoretical knowledge, the literature reveals a diverse body of established perspectives that address different dimensions of institutional development and transformation [8,9,12]. A closer comparison, however, indicates that these perspectives generally differ in their primary unit of analysis, purpose, and explanatory emphasis. Governance-oriented perspectives primarily address institutional direction, oversight, accountability, and decision-making; integrated care focuses on coordination and continuity across services and levels of care; organizational transformation emphasizes organizational change and adaptation; performance perspectives focus on measurement and achievement of objectives; organizational learning explains adaptation through learning and knowledge development; reliability perspectives emphasize resilience and dependable performance under conditions of complexity and risk; and accountability-oriented approaches link outcomes, resource use, and responsibility. Each perspective therefore provides a meaningful explanation of particular institutional dimensions, but these dimensions are not consistently specified as components of a single, interconnected institutional transformation process [8,9,12]. This distinction is important when considering established health system and organizational theories. Existing theories may incorporate multiple dimensions of organizational functioning and may recognize relationships among them; however, their primary theoretical purposes and levels of analysis vary. The conceptual gap identified in the present study is therefore not that existing frameworks fail to address institutional transformation, nor that no framework has previously recognized organizational interdependence. Rather, the gap concerns the limited explicit synthesis of multiple institutional domains within a single conceptual account in which their interactions constitute the central explanatory mechanism of institutional transformation. Accordingly, the present study does not claim that existing theories are inadequate, nor does it position IITT as a replacement for established theoretical traditions. Instead, IITT is proposed as an integrative theoretical perspective that seeks to bring relevant constructs from complementary theoretical domains into a coherent institutional systems perspective. Within this perspective, institutional transformation is conceptualized as a systemic phenomenon arising from the dynamic interaction among interconnected institutional components rather than from change within an isolated institutional function. The Tikena17-ITF represents this proposed integration by organizing these interconnected dimensions within a structured conceptual architecture. This critical synthesis therefore establishes the rationale for IITT while recognizing that its proposed integrative value remains to be demonstrated empirically. Whether the integration proposed by IITT provides explanatory or practical value beyond that offered by established theories and frameworks requires future comparative and empirical investigation.

Theoretical gap

The literature review demonstrates that research in institutional management and primary healthcare has advanced considerably over recent decades. Significant contributions have been made in institutional governance, primary healthcare, integrated care, organizational transformation, institutional performance, organizational learning, HROs, and ACOs [1,3-6,10-13]. Collectively, these bodies of knowledge have provided valuable theoretical foundations and practical guidance for improving healthcare organizations. For the purposes of this study, institutional transformation is defined as a dynamic and sustained process through which interconnected institutional structures, capabilities, relationships, processes, and outcomes are progressively reconfigured to strengthen an institution’s capacity to fulfil its mission, create healthcare value, achieve reliable performance, adapt to change, and sustain its intended functions over time. Within this definition, governance, organizational structure, institutional capabilities, care integration, performance, accountability, reliability, and sustainability are not considered synonymous with institutional transformation. Rather, they represent interconnected institutional dimensions through which transformation may occur and become observable. However, critical examination of the literature reveals that many established frameworks adopt a functional or domain-specific perspective, treating particular institutional domains as primary units of analysis. Governance frameworks primarily address governance mechanisms; performance models focus on organizational outcomes; quality frameworks emphasize quality improvement; while organizational learning, integrated care, and reliability models concentrate on their respective institutional functions [4,5,8,10-13]. Although these perspectives have generated substantial and valuable knowledge, their conceptual emphases remain differentiated. Consequently, an important unresolved question concerns not how individual institutional functions are managed, but how multiple institutional domains interact within the institution as a coherent system and contribute collectively to sustained institutional transformation. Current literature provides substantial explanations of individual domains but offers less explicit theoretical specification of the dynamic relationships among governance, institutional planning, organizational structures, institutional relationships, institutional flows, organizational culture, human capabilities, model of care, institutional performance, organizational learning, healthcare value, institutional reliability, and institutional maturity as interconnected components of a unified institutional architecture [8,13]. Accordingly, the theoretical gap identified in this study is not the absence of institutional management models or improvement frameworks. Rather, it concerns the limited theoretical integration of multiple institutional domains into a coherent explanation of institutional transformation as a system-level phenomenon. Specifically, the proposed gap relates to the need for a theoretical perspective that explains how interconnected institutional components relate to one another, how changes in one domain may influence or interact with other domains, and how these relationships may collectively shape the institution’s capacity for sustained transformation. These relationships are proposed theoretically and require empirical examination rather than being considered established causal mechanisms. To address this gap, the present study proposes the IITT as a theoretical perspective for understanding institutional transformation within primary healthcare centers. The Tikena17-ITF serves as the conceptual representation of this proposed theory by organizing its institutional components within an integrated conceptual architecture. The framework is intended to represent the structure of the institutional system and the proposed relationships among its components, including their potential contribution to institutional performance, healthcare value, institutional reliability, accountability, and institutional maturity. Therefore, the contribution of this study extends beyond the development of a conceptual framework. It proposes a theoretical perspective intended to explain institutional transformation as an integrated institutional phenomenon and to provide a conceptual foundation for future implementation models, measurement instruments, comparative studies, and empirical research. The explanatory adequacy, empirical validity, and potential added value of IITT and Tikena17-ITF remain to be established through future research.

Theory and conceptual framework

The study proposes that institutional transformation should be understood as an integrated institutional system rather than as a collection of isolated managerial initiatives or organizational functions. Based on this perspective, the IITT is developed as the explanatory theory that describes institutional transformation as an emergent systemic phenomenon arising from the continuous interaction and integration of institutional components. The Tikena17-ITF, in contrast, serves as the conceptual representation of IITT, organizing its underlying institutional dimensions into five interrelated conceptual layers comprising seventeen interconnected institutional components that represent the structural and functional architecture through which institutional transformation can be understood, examined, and subsequently tested empirically.

Integrated Institutional Transformation Theory

The principal outcome of this study is the development of the IITT, a proposed theoretical perspective for understanding institutional transformation within primary healthcare centers as a systemic phenomenon emerging from the continuous interaction of institutional components rather than from isolated managerial functions. While this integration represents a potentially novel theoretical contribution, its novelty and incremental explanatory value relative to established theories and frameworks cannot be considered empirically established at this stage. These aspects require subsequent empirical validation and comparative investigation to determine whether IITT provides explanatory value beyond existing models, a novel theoretical perspective that explains institutional transformation within primary healthcare centers as a systemic phenomenon proposed to emerge from the continuous interaction of institutional components rather than from isolated managerial functions. The theory was developed in response to the conceptual fragmentation identified across institutional governance, organizational transformation, integrated care, institutional performance, organizational learning, and healthcare value literature [3-5,10-13]. The proposed theory is founded on the assumption that healthcare organizations should be understood as integrated institutional systems in which governance, institutional planning, organizational structures, institutional relationships, institutional flows, organizational culture, human capabilities, institutional information management, model of care, institutional performance, organizational learning, healthcare value, institutional reliability, institutional accountability, institutional maturity, and institutional sustainability function as mutually dependent components of a unified institutional architecture [1,8-13]. Unlike conventional organizational models that explain institutional development through independent management domains, IITT conceptualizes institutional transformation as a theoretically proposed emergent property of institutional integration. Accordingly, the theory proposes that sustainable transformation is unlikely to be achieved by strengthening governance alone, improving organizational performance independently, redesigning models of care in isolation, or developing human capabilities separately. Rather, IITT posits that transformation may emerge through the continuous interaction and alignment of institutional components operating within an integrated institutional system [9,10,12]. These relationships are advanced as theoretical propositions of IITT and should therefore be understood as propositions to be examined and empirically tested rather than as demonstrated causal relationships. The theory further proposes that institutional performance may represent an emergent consequence of institutional integration rather than an isolated organizational objective. Likewise, healthcare value is conceptualized as the highest strategic outcome proposed to be generated through the functioning of the institutional system, while institutional reliability, accountability, institutional maturity, and long-term sustainability are regarded as potential system-level outcomes reflecting the coherence and effectiveness of institutional relationships [1,5,6,9-13]. Collectively, the IITT establishes a unified explanatory theory that synthesizes multiple institutional management traditions into a coherent institutional perspective. It provides the theoretical foundation upon which the Tikena17-ITF is subsequently developed as the conceptual representation of the proposed institutional transformation system (Figure 1).

**Figure 1:**
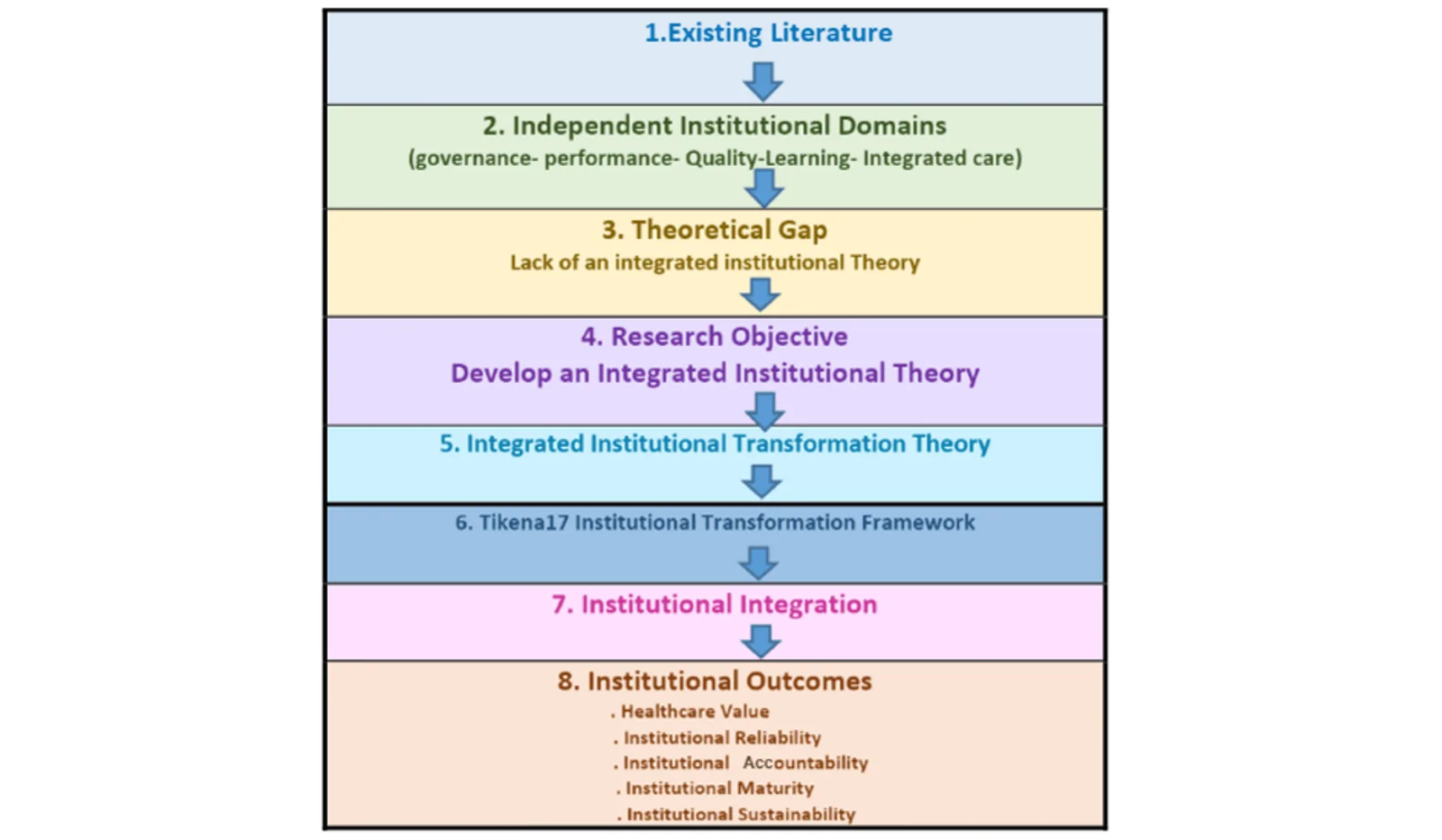
Conceptual Logic of the Integrated Institutional Transformation Theory (IITT) Conceptual logic underlying the development of the IITT, beginning with the fragmentation observed in existing institutional literature, progressing through the identified theoretical gap and research objective, and culminating in the development of the theory, its conceptual framework, institutional integration, and the strategic institutional outcomes generated by the proposed model. Figure developed by the author using Microsoft Word (Microsoft Corp., Redmond, WA, USA) and converted to a picture by Microsoft PowerPoint (Microsoft Corp.)

Theoretical propositions

Based on the conceptual synthesis undertaken in developing the IITT, nine theoretical propositions were formulated to explain the relationships among institutional integration, institutional transformation, and strategic institutional outcomes. These propositions collectively represent the theoretical core of the proposed framework and provide the foundation for future empirical validation [8,9,12]. The first proposition states that institutional transformation increases as the degree of institutional integration increases. Within the proposed theory, integration among governance, organizational structures, institutional capabilities, operational processes, and strategic outcomes constitutes the principal mechanism through which sustainable institutional transformation emerges. The second proposition identifies governance as the primary institutional driver of institutional transformation. Strategic governance establishes the institutional vision, allocates resources, coordinates institutional priorities, and aligns institutional planning with long-term transformation objectives [1,11]. The third proposition proposes that institutional capabilities-including organizational culture, workforce competencies, and institutional information management-strengthen organizational learning and innovation, thereby enhancing institutional adaptability and resilience [4,9]. The fourth proposition suggests that institutional performance should be understood as an emergent outcome of institutional integration rather than as an isolated organizational objective. Institutional performance therefore reflects the coherence and interaction of institutional components operating as a unified system [8,9,12]. The fifth proposition identifies healthcare value as the ultimate strategic outcome generated by institutional transformation. Rather than measuring isolated organizational outputs, healthcare value reflects the integrated performance of the institutional system in delivering effective, efficient, patient-centered, and sustainable healthcare services [1,3,10]. The sixth proposition proposes that institutional reliability develops through the integration of governance, organizational processes, learning systems, and operational performance. Reliable institutions consistently deliver safe, resilient, and dependable healthcare services under varying organizational conditions [4,5]. The seventh proposition emphasizes that institutional accountability sustains long-term institutional transformation by strengthening transparency, organizational trust, leadership responsibility, and continuous institutional evaluation [10,11]. The eighth proposition proposes that institutional maturity represents the cumulative result of progressive institutional development across all organizational domains. Mature institutions demonstrate integrated governance, adaptive organizational capability, continuous learning, and sustainable institutional performance. Finally, the ninth proposition states that institutional sustainability depends upon the continuous interaction among institutional components rather than the optimization of individual organizational functions. Sustainable institutional development therefore requires maintaining dynamic balance across governance, institutional architecture, organizational capability, operational transformation, and strategic outcomes [1,3,4,9]. Collectively, these propositions provide the first theoretical structure derived from the IITT and establish a coherent basis for future hypothesis testing, framework validation, institutional maturity assessment, and comparative organizational research (Table 1).

**Table 1:** Theoretical Propositions of the Integrated Institutional Transformation Theory (IITT) This table summarizes the nine theoretical propositions derived from the IITT. Each proposition describes a fundamental institutional relationship proposed by the theory and identifies its corresponding expected institutional outcome. Together, these propositions constitute the theoretical foundation for future empirical testing, institutional maturity assessment, and validation of the Tikena17 Institutional Transformation Framework (Tikena17-ITF).

Proposition	Statement	Expected institutional outcome
P1	Institutional transformation increases as the level of integration among institutional components increases	Sustainable institutional transformation
P2	Institutional governance serves as the primary strategic driver influencing all other institutional components	Strategic alignment
P3	Institutional capabilities strengthen organizational learning, innovation, and evidence-informed decision-making	Adaptive institutional capability
P4	Institutional performance emerges from the integration of institutional components rather than isolated organizational functions	Organizational effectiveness
P5	Healthcare value represents the ultimate strategic outcome of the institutional system	Sustainable healthcare value creation
P6	Institutional reliability results from the integration of governance, risk management, organizational learning, and institutional performance	Patient safety and organizational resilience
P7	Institutional accountability sustains institutional transformation through transparency, continuous improvement, and organizational trust	Institutional trust and accountability
P8	Institutional maturity reflects the developmental level of the institutional system as a whole rather than individual institutional domains	Organizational maturity
P9	Institutional sustainability depends upon the continuous dynamic interaction among institutional components	Long-term institutional sustainability

Tikena17 Institutional Transformation Framework

From Theory to Conceptual Framework

The IITT provides the theoretical explanation of institutional transformation as a systemic phenomenon emerging from the continuous interaction among institutional components. However, theoretical explanation alone is insufficient for practical application. A conceptual representation is required to translate theoretical propositions into an institutional architecture that can be understood, implemented, measured, and empirically examined. Accordingly, the Tikena17-ITF is developed as the conceptual representation of IITT. The framework organizes the institutional transformation into an integrated conceptual architecture that explains the structural and functional relationships governing institutional transformation within primary healthcare centers. Tikena17-ITF should not be interpreted as an independent management methodology or implementation model. Rather, it represents the conceptual architecture through which the theoretical assumptions of IITT are translated into interconnected institutional components capable of supporting implementation models, measurement instruments, and institutional transformation programs.

Conceptual architecture of the framework

The Tikena17-ITF conceptualizes primary healthcare as an integrated institutional system composed of 17 interconnected institutional components. Together, these components represent the institutional architecture required to enable coherent, sustainable, and system-wide transformation. The 17 components are organized into five interrelated conceptual layers, each representing a distinct systemic function while remaining dynamically dependent on, and interacting with, the other layers.

Layer One: Institutional Strategic Direction

Governance and institutional planning: This layer establishes the institutional strategic direction through governance, institutional planning, strategic priorities, decision-making structures, and organizational purpose. It provides the overarching direction and institutional intent that guide the design, integration, and transformation of the organization.

Layer Two: Institutional Architecture

Organizational structures, institutional relationships, and institutional flows: This layer constitutes the structural and connective foundation of institutional integration. Organizational structures define the distribution of responsibilities, authority, roles, and functions; institutional relationships establish the connectivity and coordination among organizational units, professional groups, and institutional functions; and institutional flows enable the movement and alignment of information, decisions, resources, and processes across the institution.

Together, these components create the connective architecture through which organizational functions interact as an integrated institutional system. Thus, institutional integration emerges not merely from the presence of organizational structures, but from the coherence, connectivity, and interaction established through institutional relationships and flows.

Layer Three: Institutional Capabilities

Institutional culture, human capabilities, and institutional information management: This layer provides the organizational, human, intellectual, cultural, and informational capabilities required to operate, learn, adapt, and continuously develop the institution. It enables organizational learning, knowledge creation, innovation, evidence-informed decision-making, and the development of institutional capacity.

Layer Four: Institutional Operational Transformation

Model of care, institutional performance, institutional learning, and quality and institutional excellence: This layer translates institutional direction, architecture, and capabilities into integrated operational transformation. It connects the model of care with institutional performance, continuous institutional learning, quality improvement, and institutional excellence, thereby converting institutional capacity into measurable and sustainable operational performance.

Layer Five: Institutional Strategic Outcomes

Healthcare value, institutional reliability, institutional accountability, institutional maturity, and institutional sustainability: This layer represents the strategic outcomes emerging from the functioning and integration of the institutional system. These outcomes reflect the institution’s capacity to generate sustainable healthcare value, maintain institutional reliability, demonstrate accountability, achieve institutional maturity, and sustain performance and development over time.

Systemic relationship among the layers

The five layers should not be interpreted as a strictly linear sequence. Institutional Strategic Direction shapes the institutional architecture, Institutional Architecture provides the structural and connective foundation through which institutional integration occurs, and this integration enables the effective mobilization of Institutional Capabilities and Institutional Operational Transformation. Institutional Strategic Outcomes consequently emerge from the continuous interaction among the layers rather than from any single layer operating independently. Accordingly, the framework represents a dynamic and integrated institutional system in which relationships, flows, interactions, and feedback mechanisms continuously connect strategic direction, institutional architecture, capabilities, operations, and outcomes. This systemic configuration reflects the central proposition of IITT that institutional transformation is an emergent phenomenon arising from the continuous interaction and integration of institutional components rather than from the isolated improvement of individual functions.

The framework as the conceptual representation of IITT

Tikena17-ITF represents the conceptual and operational manifestation of the IITT. It introduces no additional theoretical assumptions beyond those established by IITT; instead, it translates theoretical principles into an integrated institutional architecture suitable for practical implementation. Accordingly, the framework provides the conceptual foundation for developing institutional transformation programs, implementation methodologies, diagnostic instruments, institutional maturity assessment models, and empirical studies designed to evaluate the theoretical propositions of IITT. Within this perspective, the relationship between IITT and Tikena17-ITF is complementary: the theory explains the phenomenon of institutional transformation, while the framework provides its structured conceptual representation for implementation, measurement, and scientific validation (Figure 2).

**Figure 2:**
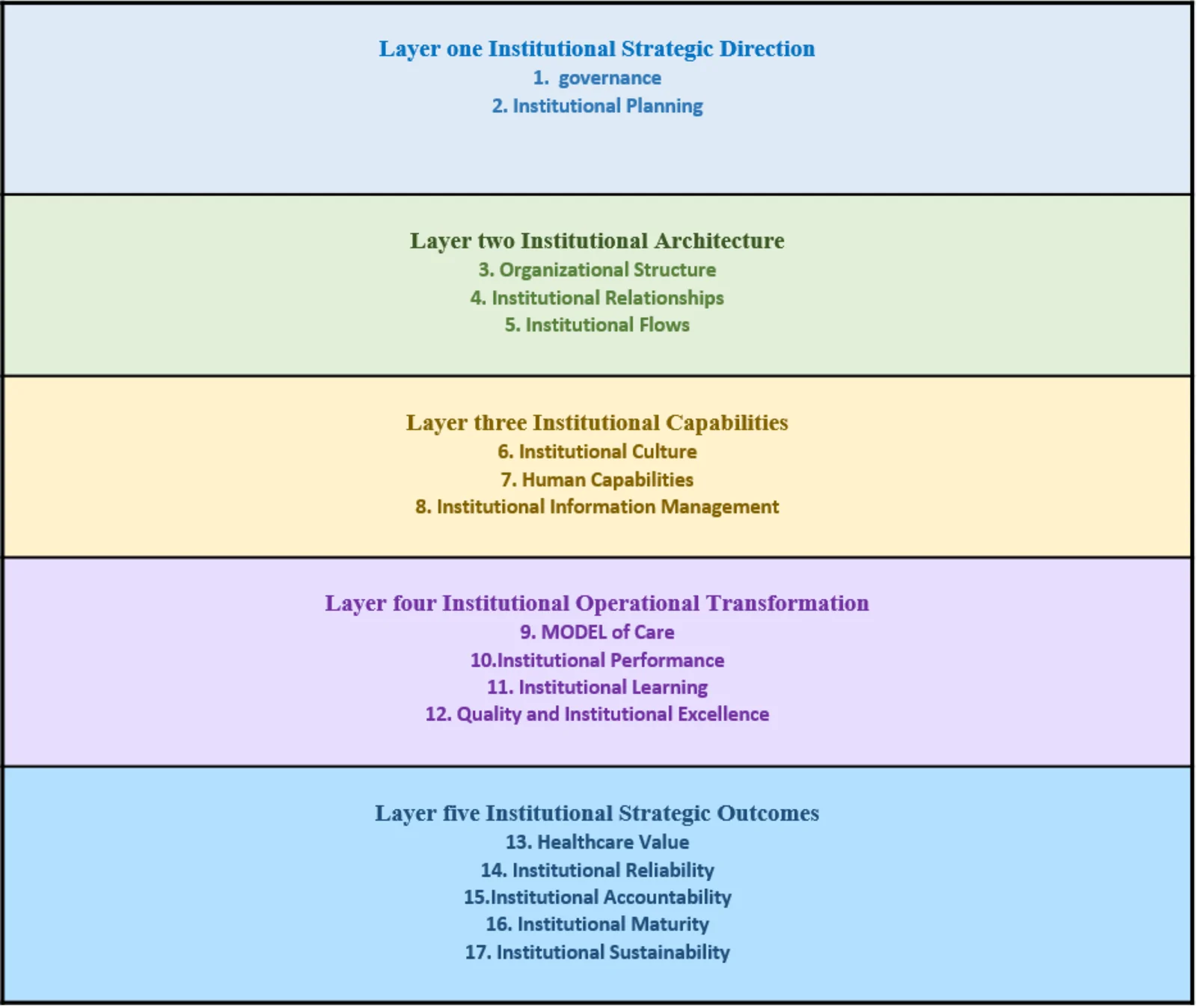
Tikena17 Institutional Transformation Framework (Tikena17-ITF) The Tikena17-ITF is an integrated institutional transformation framework comprising five interconnected layers and 17 interdependent components that collectively explain how an institution moves from institutional strategic direction to institutional strategic outcomes. The five layers are vertically integrated, while their components interact horizontally through dynamic institutional relationships, flows, feedback, and learning, allowing the institution to continuously adapt and transform. Thus, Tikena17-ITF represents a coherent transformation system in which direction shapes architecture, architecture enables capabilities, capabilities drive operational transformation, and operational transformation generates sustainable institutional strategic outcomes. Figure developed by the author using Microsoft Word (Microsoft Corp., Redmond, WA, USA) and converted to a picture by Microsoft PowerPoint (Microsoft Corp.)

## Discussion

Interpretation of the findings

The present study introduces the IITT as a unified theoretical explanation for institutional transformation in primary healthcare centers. Unlike existing institutional and healthcare management frameworks, which primarily examine governance, organizational performance, integrated care, organizational learning, healthcare quality, or patient safety as relatively independent domains, the proposed theory conceptualizes these elements as interconnected components of a single institutional system [1-6,9,12,13]. This integrated perspective addresses a long-standing limitation within healthcare management literature. Although numerous frameworks have contributed substantially to improving individual organizational domains, relatively few have attempted to explain how these institutional components interact dynamically to produce sustainable organizational transformation [4,8,9,12,13]. However, the interpretation of IITT should also recognize that its proposed domains are not entirely distinct from existing theoretical traditions. Governance, organizational learning, integrated care, quality, reliability, and performance have each been extensively addressed by established frameworks [1-6,9,12,13]. The contribution of IITT therefore lies less in introducing these domains individually and more in proposing that they can be interpreted as interdependent elements of a broader institutional transformation system. In this respect, IITT overlaps conceptually with several existing perspectives, while seeking to provide a higher-order explanation of how these domains may interact within primary healthcare institutions. Whether this integration provides explanatory value beyond existing frameworks remains an empirical question. The findings further support the theoretical proposition that institutional transformation may be associated with the degree of coherence and interaction among institutional components. Consequently, institutional transformation has often been implemented through fragmented improvement initiatives that optimize individual functions without adequately strengthening institutional coherence. The IITT extends institutional theory by proposing that sustainable transformation may emerge from continuous interaction among governance, institutional architecture, organizational capabilities, operational transformation, and strategic institutional outcomes [1,4,8-13]. However, this proposed relationship should not be interpreted as evidence that institutional integration alone produces transformation. Alternative explanations may include leadership effectiveness, organizational resources, workforce characteristics, organizational culture, technological development, external regulatory requirements, policy changes, and broader health system reforms. These factors may independently influence institutional transformation or may modify the relationship proposed by IITT. Similarly, the interpretation of organizational performance as an emergent institutional property represents a theoretical proposition rather than a demonstrated causal relationship. Rather than viewing organizational performance as an independent managerial objective, IITT proposes that performance may emerge from the interaction and alignment of institutional components. Likewise, healthcare value, institutional reliability, accountability, institutional maturity, and sustainability are interpreted as potential cumulative outcomes of institutional coherence rather than isolated organizational achievements [1,3-6,9-13]. These propositions require empirical investigation to determine whether institutional integration is associated with improved performance and whether the proposed system-level outcomes can be distinguished empirically from the constructs already addressed within established organizational and healthcare frameworks. The applicability of IITT may also depend on specific contextual conditions. The proposed relationships may vary according to organizational size, institutional maturity, governance arrangements, resource availability, degree of organizational autonomy, workforce characteristics, and the wider regulatory and health system environment. Because the theory was developed specifically for primary healthcare centers, its applicability to hospitals, specialized healthcare organizations, or broader health system transformation should not be assumed without further empirical examination. These boundary conditions are particularly important because institutional integration may have different meanings and effects across organizations with different levels of complexity and autonomy. The development of the Tikena17-ITF further translates the theoretical model into a practical institutional architecture. Unlike many conceptual healthcare frameworks that focus on specific operational dimensions, Tikena17 provides an integrated organizational roadmap linking strategic governance with institutional structures, organizational capabilities, healthcare delivery, and long-term institutional outcomes. This layered architecture enables healthcare organizations to visualize transformation as a progressive institutional process rather than as a collection of disconnected management initiatives. Nevertheless, Tikena17-ITF should be understood as the conceptual representation of IITT rather than as empirical validation of the theory. Its practical usefulness, as well as the validity of the relationships represented within its five conceptual layers, requires subsequent empirical assessment. Overall, the interpretation of the findings suggests that IITT offers a coherent integrative perspective for understanding institutional transformation in primary healthcare, while also acknowledging substantial areas of conceptual overlap with established frameworks. Its central proposition is that institutional transformation may be better understood through the interaction and alignment of institutional components than through isolated organizational interventions. At this stage, however, this proposition should be regarded as theoretically plausible rather than empirically established. Future research should therefore examine alternative explanations, test the proposed relationships across different primary healthcare contexts, determine whether institutional integration provides explanatory value beyond existing frameworks, and identify the organizational and contextual conditions under which the proposed theory is most applicable.

Relationship to existing literature

The proposed theory complements rather than replaces existing healthcare management frameworks. Clinical governance continues to explain institutional accountability and quality assurance [11,12], integrated care strengthens coordination across healthcare services [3,10], learning organization theory explains organizational adaptation and knowledge creation [4], while HRO theory contributes to understanding organizational resilience and patient safety [5,13]. These perspectives therefore overlap with several elements incorporated within IITT, particularly in relation to governance, coordination, learning, reliability, and quality. However, they generally approach these domains from distinct theoretical or functional perspectives. The contribution of IITT is consequently not the introduction of entirely new institutional domains, but the proposition that these established domains can be understood as interconnected components of a broader institutional transformation system. From this perspective, the IITT provides a higher-order institutional perspective that seeks to connect these established theoretical traditions within a single explanatory model. Nevertheless, this integrative claim should be interpreted cautiously. Existing systems-oriented approaches may already provide mechanisms for explaining organizational interdependence, and the distinction between IITT and these approaches will ultimately depend on whether empirical testing demonstrates explanatory relationships that are not adequately captured by existing theories. Thus, IITT should be viewed as an integrative theoretical proposition rather than as a replacement for established frameworks. Its potential contribution lies in synthesizing complementary organizational perspectives while preserving their individual theoretical contributions and examining how their interaction may relate to institutional transformation.

An additional proposition of IITT is that institutional integration may constitute an important mechanism through which sustainable transformation occurs. However, alternative explanations should also be considered. Institutional transformation may be influenced by factors such as leadership, organizational resources, external policy and regulatory pressures, workforce characteristics, technological change, organizational culture, or broader health system reforms. These factors may independently influence transformation or may moderate the relationship between institutional integration and transformation. Accordingly, the theory does not establish that integration alone produces transformation; rather, it proposes that the interaction and alignment of institutional components may contribute to transformation within particular organizational and health system contexts. This distinction is important because the relative contribution of institutional integration compared with other determinants remains an empirical question. The applicability of IITT may also be subject to important boundary conditions. The proposed relationships may differ according to the size and complexity of the healthcare organization, its governance structure, maturity level, available resources, degree of autonomy, and the characteristics of the surrounding health system. The theory was developed specifically in the context of primary healthcare, and therefore, its explanatory relevance to hospitals, specialized healthcare organizations, or broader health system transformation should not be assumed without further investigation. Similarly, the proposed relationships may operate differently across health systems with substantially different regulatory, organizational, and financing arrangements. These contextual conditions should therefore be considered in future empirical testing. The theory also has an important limitation at its current stage of development. The relationships proposed by IITT, including the proposition that institutional performance may emerge from institutional integration, have not yet been empirically validated. The apparent coherence of the framework therefore should not be interpreted as evidence of causality or predictive validity. Empirical studies will be required to determine whether greater institutional integration is associated with improved transformation outcomes, whether institutional integration adds explanatory value beyond existing organizational theories, and whether the proposed relationships remain consistent across different primary healthcare settings.

This systems-oriented interpretation aligns with contemporary organizational systems theory while extending its application specifically to institutional transformation in primary healthcare [9]. Nevertheless, this extension should be considered a theoretical proposition rather than an established theoretical distinction. The extent to which IITT provides explanatory value beyond existing systems, governance, integrated care, learning, and high-reliability perspectives remains to be determined through comparative and empirical research. Overall, the discussion supports the proposition that primary healthcare transformation may be better understood as the coordinated evolution of an integrated institutional system rather than solely as a sequence of isolated organizational improvements. However, this proposition should be considered alongside alternative explanations, contextual conditions, and areas of theoretical overlap with established frameworks. The principal contribution of IITT at this stage is therefore the provision of a coherent integrative hypothesis that can be empirically examined, refined, or potentially revised through future research. This establishes a foundation for empirical testing, institutional assessment, organizational redesign, and health system reform initiatives without assuming that the proposed relationships have already been demonstrated.

Scientific contribution

The IITT contributes to healthcare management literature by introducing a comprehensive institutional perspective that unifies previously fragmented theoretical domains into a single explanatory framework. Existing healthcare theories have significantly advanced knowledge in governance, organizational learning, integrated care, healthcare quality, institutional performance, and patient safety; however, they have generally evolved as independent conceptual traditions with limited integration across institutional boundaries [1-6,9-13]. The principal scientific contribution of IITT lies in conceptualizing primary healthcare organizations as integrated institutional systems in which institutional transformation emerges from the continuous interaction of governance, organizational structures, institutional capabilities, operational transformation, and strategic institutional outcomes. This systems-based interpretation extends traditional institutional theory beyond static organizational structures toward a dynamic institutional transformation model capable of explaining how multiple organizational components collectively generate sustainable institutional development [9]. Furthermore, the development of the Tikena17-ITF provides an operational conceptual architecture that translates the theoretical principles of IITT into a structured institutional model suitable for organizational assessment, institutional redesign, and future empirical validation. The accompanying theoretical propositions establish a coherent research agenda that enables future quantitative, qualitative, and mixed-method investigations to examine institutional transformation across diverse primary healthcare contexts. Collectively, IITT represents a higher-order institutional theory that complements existing healthcare management frameworks while providing a unified conceptual foundation for future institutional transformation research.

Practical implications

Beyond its theoretical contribution, the IITT offers practical implications for healthcare leaders, policymakers, and organizational managers responsible for strengthening primary healthcare institutions. Rather than implementing isolated organizational improvement initiatives, healthcare organizations may utilize the proposed framework to evaluate institutional integration across governance, organizational structures, institutional capabilities, healthcare delivery processes, and strategic institutional outcomes [10-13]. The Tikena17-ITF can serve as a practical institutional roadmap for organizational diagnosis, strategic planning, institutional maturity assessment, and long-term transformation programs. By identifying institutional strengths, weaknesses, and interaction patterns across its 17 institutional components, healthcare organizations may prioritize integrated institutional development rather than fragmented organizational reforms [9,12]. For policymakers, the theory provides a conceptual basis for designing institutional transformation strategies that align governance reforms, workforce development, organizational learning, healthcare quality, and performance management within a coherent institutional system [1-5,9]. Similarly, healthcare executives may employ the framework to strengthen institutional reliability, accountability, and sustainability while supporting continuous organizational adaptation in increasingly complex healthcare environments [4,5,9]. Ultimately, the practical value of IITT lies in shifting institutional transformation from isolated managerial interventions toward an integrated systems approach capable of supporting sustainable organizational performance and long-term healthcare value creation.

Limitations

This study has several limitations that should be considered when interpreting its theoretical and conceptual contribution. First, IITT and the Tikena17-ITF are conceptually derived from the synthesis and integration of existing theoretical and empirical literature and have not yet undergone empirical validation. Accordingly, the proposed relationships among institutional components should be regarded as theoretical propositions rather than demonstrated causal relationships.

Second, the development of the theory and framework may be influenced by literature-selection bias, as the conceptual synthesis reflects the scope, availability, and interpretation of the literature selected for this study. Although the framework integrates multiple institutional domains, relevant perspectives or alternative theoretical explanations may not have been fully represented.

Third, the applicability and transferability of IITT and Tikena17-ITF across different healthcare settings have not yet been established. The framework was developed primarily in the context of primary healthcare centers, and its relevance to hospitals, specialized healthcare organizations, or other healthcare delivery settings requires further investigation.

Finally, the proposed theory and framework have not yet demonstrated their applicability across different health system contexts, particularly where governance structures, financing mechanisms, regulatory environments, organizational autonomy, and institutional maturity differ substantially. Future empirical research should therefore test the proposed relationships, assess the framework across diverse healthcare and health system settings, and examine its incremental explanatory and practical value relative to established models.

## Conclusions

This study introduces the IITT as a proposed theoretical perspective for understanding institutional transformation in primary healthcare centers as an integrated and systemic phenomenon. Rather than viewing transformation as the cumulative result of improving separate institutional functions, IITT conceptualizes it as potentially emerging from the dynamic relationships and interactions that constitute the institution as a whole. The Tikena17-ITF provides the conceptual representation of this theoretical perspective and translates its central assumptions into an integrated institutional structure. The contribution of the study therefore lies in proposing a theoretical and conceptual basis for understanding institutional transformation as a distinct institutional phenomenon, while recognizing that the explanatory relationships proposed by IITT and the practical utility of Tikena17-ITF remain to be empirically examined. The theory and framework provide hypotheses and a foundation for further theoretical development, empirical testing, and potential practical application. Future research should examine the proposed theoretical relationships, assess the incremental explanatory value of IITT relative to established frameworks, and determine the applicability of Tikena17-ITF across different primary healthcare and institutional contexts. In this sense, IITT and Tikena17-ITF represent promising initial models for advancing an integrated understanding of institutional transformation and for exploring how institutional integration may relate to the development of more resilient, reliable, accountable, effective, and sustainable healthcare organizations.
